# A Critical Perspective on Photothermal De‐Icing

**DOI:** 10.1002/adma.202415237

**Published:** 2024-12-23

**Authors:** Siyan Yang, Jiazheng Liu, Muhammad Jahidul Hoque, Anxu Huang, Yiyang Chen, Wentao Yang, Jie Feng, Nenad Miljkovic

**Affiliations:** ^1^ Department of Mechanical Science and Engineering The Grainger College of Engineering University of Illinois Urbana‐Champaign Urbana IL 61801 USA; ^2^ Materials Research Laboratory University of Illinois at Urbana‐Champaign Urbana IL 61801 USA; ^3^ Department of Electrical and Computer Engineering The Grainger College of Engineering University of Illinois at Urbana‐Champaign Urbana IL 61801 USA; ^4^ Institute for Sustainability Energy and Environment University of Illinois at Urbana‐Champaign Urbana IL 61801 USA; ^5^ International Institute for Carbon Neutral Energy Research (WPI‐I2CNER) Kyushu University 74 Motooka Nishi‐ku Fukuoka 819‐0395 Japan

**Keywords:** de‐icing, ice adhesion, photothermal

## Abstract

To tackle the formidable challenges posed by extreme cold weather events, significant advancements have been made in developing functional surfaces capable of efficiently removing accreted ice. Nevertheless, many of these surfaces still require external energy input, such as electrical power, which raises concerns regarding their alignment with global sustainability goals. Over the past decade, increasing attention has been directed toward photothermal surface designs that harness solar energy−a resource available on Earth in quantities exceeding the total reserves of coal and oil combined. By converting solar energy into heat, these designs enable the transformation of the interfacial solid‐solid contact (ice‐substrate) into a liquid‐solid contact (water‐substrate), significantly reducing interfacial adhesion and facilitating rapid ice removal. This critical perspective begins by emphasizing the advantages of photothermal design over traditional de‐icing methods. It then delves into an in‐depth analysis of three primary photothermal mechanisms, examining how these principles have expanded the scope of de‐icing technologies and contributed to advancements in photothermal surface design. Finally, key fundamental and technical challenges are identified, offering strategic guidelines for future research aimed at enabling practical, real‐world applications.

## Graphical abstract

This perspective provides a comprehensive overview of three distinct photothermal mechanisms. It discusses recent advancements in photothermal de‐icing and elucidates key challenges in their application. Through a detailed presentation of a comparative dataset and critical insights, valuable guidance is provided for future research, particularly in the strategic selection of materials and structure designs, to advance practical de‐icing solutions in real‐world applications.

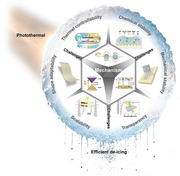

## 1 Introduction

The need for effective de‐icing strategies to combat cold weather has been a persistent challenge throughout human civilization. Conventional active de‐icing efforts include electro‐thermal,^[^ ^1^ ^]^ pulse electro‐thermal,^[^ ^2^ ^]^ ultrasonic,^[^ ^3^ ^]^ mechanical,^[^ ^4^ ^]^ and chemical approaches,^[^ ^5^ ^]^ which aim to melt, shake, blow, or scrape off accumulated ice or lower its freezing point.^[^ ^6^ ^]^ While effective, these active approaches are hindered by significant drawbacks such as high energy input, complicated setups, meticulous maintenance requirements, and limited environmental sustainability.^[^ ^7^ ^]^ These limitations highlight the urgent need for a paradigm shift in the development of functional de‐icing materials, especially given the increasing frequency and severity of extreme weather events.^[^ ^8^ ^]^ In response, passive de‐icing strategies, which rely on the inherent properties of functional materials, have gained attention. Common examples include superhydrophobic surfaces,^[^ ^9^ ^]^ lubricant‐infused surface,^[^ ^10^ ^]^ polymer brush or gels,^[^ ^11^ ^]^ low‐modulus elastomers,^[^ ^12^ ^]^ low interfacial‐toughness materials,^[^ ^13^ ^]^ or suspended thin metallic surface.^[^ ^14^ ^]^ These approaches leverage mechanisms such as reducing surface energy and contact area,^[^ ^9^ ^]^ creating liquid‐like slippery interface,^[^ ^10^, ^11^ ^]^ or initiating interfacial cavitation, cracks, or buckling.^[^ ^9^, ^10^, ^11^, ^13^, ^14^ ^]^ Despite their innovative design, most passive methods still require some level of energy input to facilitate de‐icing,^[^ ^15^ ^]^ which stands in stark contrast to global priorities for sustainability, carbon neutrality, and energy efficiency.

Toward this end, remarkable research efforts have been put to the exploitation of solar energy, a resource celebrated for its inherently green and sustainable nature. With an immense amount of energy (105 × 109 TWh) absorbed by Earth's surface−exceeding the combined reserves of coal and oil.^[^ ^16^ ^]^ Solar energy can be harnessed and converted into diverse energy forms, including electricity, chemical fuels, and thermal energy, facilitated by the underlying photovoltaic, photochemical, and photothermal processes, respectively.^[^ ^17^ ^]^ Among these, the photothermal process stands out for its ability to directly convert solar energy into thermal energy, achieving the highest conversion efficiency. This photothermal paradigm has found wide‐ranging applications, from distillation to steam generation,^[^ ^18^ ^]^ desalination,^[^ ^19^ ^]^ and de‐icing.^[^ ^20^ ^]^ Specifically, in de‐icing applications, the solar‐to‐heat property enables rapid heating of material surfaces to temperatures above the melting point of ice. This process transforms the solid‐solid interface (ice‐substrate) into a liquid‐solid interface (water‐substrate), allowing bulk ice to easily slide off under gravity.^[^ ^21^ ^]^ Even in cases where meltwater is not completely shed, continuous surface heating can promote evaporation, leaving behind a dry surface.^[^ ^11^, ^22^ ^]^ As such, photothermal designs demonstrate great promise in achieving effective de‐icing across a wide range of ice sizes, from microscale to macroscale.

In this perspective, we begin by exploring the rationale underlying adopting photothermal design as a viable approach to achieve effective de‐icing. This is followed by an in‐depth analysis of three core photothermal mechanisms inherent to nanomaterials. Building on this foundational understanding, we uncover latest advancements in translating these principles into innovative designs that intricately integrate material interfaces with sophisticated structures, aiming to achieve high‐efficiency de‐icing performance. Finally, we engage in a critical discourse surrounding the key fundamental and technical obstacles that persist, to guide future research efforts toward the realization of practical, real‐world de‐icing technologies. This perspective aims to provide the scientific community with a deeper understanding of the nuanced photothermal properties of diverse materials and offers strategic direction for the design of photothermal nanomaterials, facilitating their application in a wide range of real‐world de‐icing scenarios.

## 2 The Rationale of Choosing the Photothermal Approach

**Figure 1:**
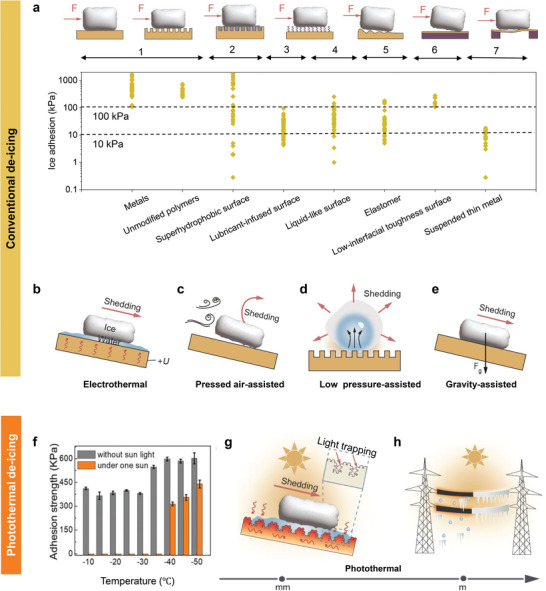
Comparison of conventional and photothermal de‐icing techniques. a) Ice adhesion strength reported in the past literature for various surface designs, along with the corresponding working mechanisms shown above. For a summary of all data, please see Table S1 (Supporting Information).^[^ ^9^, ^10^, ^11^, ^12^, ^13^, ^14^, ^23^ ^]^ Error bars for each data are not shown for clarity. Schematics showing typical passive de‐icing approaches: b) electro‐thermal approach,^[^ ^15^, ^26^ ^]^ c) air‐induced shear,^[^ ^27^ ^]^ d) low pressure driven self‐lodging,^[^ ^28^ ^]^ e) gravity driven ice shedding.^[^ ^24^ ^]^ f) Comparison in ice adhesion strength between non‐photothermal and photothermal approaches. Photothermal‐assisted interfacial ice melting and its advantage in ice shedding from g) small‐scale^[^ ^32^ ^]^ to h) large‐scale applications. Schematics not to scale. d) Adapted under the terms of the CCBY Creative Commons Attribution 4.0 International license.^[^ ^28^ ^]^ Copyright 2023, The Authors, published by Springer Nature. f) Adapted with permission.^[^ ^31^ ^]^ Copyright 2023, Springer Nature. h) Adapted with permission.^[^ ^33^ ^]^ Copyright 2022, Wiley‐VCH GmbH.

**Figure** **1**a provides a comprehensive overview of typical de‐icing approaches that leverage the rigid nature of ice and the intrinsic properties of materials to passively facilitate de‐icing.^[^ ^9^, ^10^, ^11^, ^12^, ^13^, ^14^, ^23^ ^]^ Despite the diversity of these methods, they share a common requirement–achieving low interfacial ice adhesion, defined as τ_ice_ = *F*/*S*_ice_,^[^ ^24^ ^]^ where *F* denotes the shear force required to detach an iced area, *S*_ice_. Meeting this seemingly straightforward criterion, however, presents several challenges. One challenge is that a large body of surface designs, particularly those using pristine material surfaces (Figure 1a; full data in Table S1, Supporting Information^[^ ^9^, ^10^, ^11^, ^12^, ^13^, ^14^, ^23^ ^]^), exhibit ice adhesion values exceeding 100 kPa. This value is widely recognized as the threshold for low ice adhesion.^[^ ^25^ ^]^ This challenge becomes more pronounced when attempting to achieve de‐icing by the weight of the accumulated ice alone, which corresponds to a required ice adhesion value of 10 kPa.^[^ ^11^, ^14^, ^24^ ^]^ This is because the ice adhesion of most surfaces, even for superhydrophobic surfaces that boast a negligible ice‐substrate contact area and low surface energy,^[^ ^23^ ^]^ exceeds this metric. Consequently, these designs require external energy input or rigorous environmental conditions, such as electrical heating,^[^ ^15^, ^26^ ^]^ pressed air blowing,^[^ ^27^ ^]^ or ultra‐low environment pressure,^[^ ^28^ ^]^ to aid in ice melting, evaporation, or detachment (Figure 1b–d).

Challenges persist even for surface designs with ice adhesion below the 10 kPa threshold, as illustrated in Figure 1e. For instance, lubricant‐infused slippery surfaces and liquid‐like molecular brush surfaces lose efficacy over time due to lubricant depletion or medium degradation,^[^ ^29^ ^]^ which in turn leads to a substantial increase in ice adhesion. Similarly, elastomer‐based and low‐interfacial‐toughness surfaces, while promising, suffer from limited mechanical durability for long‐term use and are effective only for bulk ice with lateral sizes exceeding 10 cm.^[^ ^12^, ^24^, ^30^ ^]^ Recent findings indicate that thin metallic surfaces can reduce interfacial adhesion through surface buckling.^[^ ^14^ ^]^ Although this approach is robust and simplifies manufacturing, it requires suspension on elastomer substrates, which significantly increases system size.

As an alternative, photothermal surface designs promise to address these concerns. By utilizing efficient solar‐to‐heat conversion, the photothermal effect induces interfacial ice melting, transitioning the system from a sticky solid‐solid (ice‐substrate) interface to a non‐slippery liquid‐solid (water‐substrate) interface. In this scenario, the previously undesired water film becomes a lubricating layer, dramatically reducing interfacial ice adhesion−potentially to 0 kPa, even at sub‐freezing temperatures as low as −40 °C (Figure 1f).^[^ ^31^ ^]^ Given that most industrial facilities operate above −40 °C and the versatility of photothermal de‐icing across a wide range of ice thicknesses and lateral sizes−from millimeter‐scale droplets (Figure 1g)^[^ ^32^ ^]^ to meter‐scale ice on transmission cables and wind turbine blades (Figure 1h),^[^ ^33^ ^]^ this approach merits primary consideration.

## 3 Fundamental Mechanisms Governing Photothermal Effect

**Figure 2:**
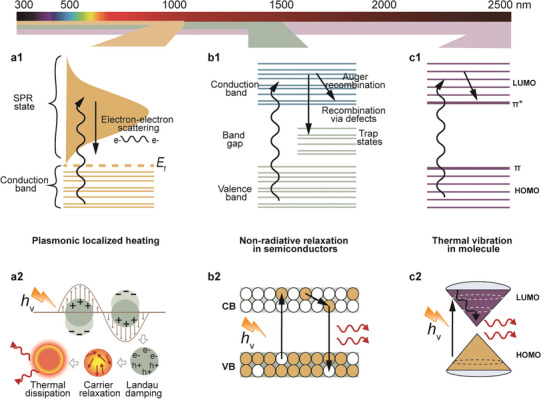
Mechanisms governing the three typical photothermal effects. a) Plasmonic localized heating.^[^ ^35^ ^]^ b) Non‐radiative relaxation.^[^ ^35^ ^]^ c) Thermal vibration in molecules.^[^ ^35^ ^]^ a1‐c1) Adapted with permission.^[^ ^35^ ^]^ Copyright 2019, the Royal Society of Chemistry. a2) Adapted with permission.^[^ ^39^ ^]^ Copyright 2019, Elsevier; Copyright 2014, Springer. b2) Adapted with permission.^[^ ^41^ ^]^ Copyright 2017, Wiley‐VCH GmbH. c2) Adapted with permission.^[^ ^38^ ^]^ Copyright 2020, Springer. The color band on top denotes the wavelength of excitation where each photothermal heating mode is dominant. Schematics not to scale.

Optimizing the photothermal effect of materials necessitates a comprehensive understanding of the underlying photothermal conversion mechanisms. Fundamentally, the photothermal effect stems from photoexcitation, which leads to the partial or full generation of heat. When the energy of an incident photon matches the energy gap between two molecular energy levels, the photon can be absorbed by the material, promoting an electron from the ground state to an excited state. These excited molecules, being inherently unstable, typically relax back to the ground state via either radiative or non‐radiative deactivation pathways. In the radiative deactivation process, the excited‐state molecule emits a photon, resulting in the phenomenon of fluorescence or phosphorescence.^[^ ^34^ ^]^ Conversely, the non‐radiative deactivation process releases the excess energy as heat, with variations in the photothermal effect depending on the material.^[^ ^34^ ^]^ As illustrated in **Figures** **2**a1‐c1, absorbed light can initiate various mechanisms that ultimately dissipate energy as heat. One such mechanism is the generation of localized surface plasmon resonance (LSPR) in free electrons, referred to as plasmonic localized heating.^[^ ^35^ ^]^ Additionally, absorbed light can excite bound electron‐hole pairs through electronic transitions on semiconductor surfaces, a process known as non‐radiative relaxation.^[^ ^35^, ^36^ ^]^ In π‐conjugated systems characteristic of carbon‐based materials, the absorbed light induces π‐π* transitions, leading to the thermal vibration of molecules.^[^ ^35^, ^37^ ^]^ Collectively, these light‐induced processes convert the absorbed energy into heat, forming the basis of photothermal effects.

Plasmonic localized heating occurs when incident light induces LSPR in free electrons, typically on metallic nanostructures. During this process, as depicted in Figure 2a2, the surfaces of the metallic nanostructures undergo LSPR, which alters the local energy flow density around the nanoparticles and enhances interactions between the optical field and charge carriers.^[^ ^35^, ^38^ ^]^ The LSPR relaxes through non‐radiative Landau damping, generating high‐energy electron‐hole pairs.^[^ ^39^ ^]^ Initially, the energy distribution among these carriers is highly non‐uniform; however, electron‐electron scattering dominates, redistributing the energies and producing “hot electrons” and “hot holes” with energy levels exceeding thermal excitation at ambient temperatures. This energy is then transferred to the lattice through electron‐phonon scattering, elevating the local lattice temperature.^[^ ^40^ ^]^ Finally, phonon‐phonon scattering gradually dissipates the thermal energy into the surrounding environment.

The non‐radiative relaxation process normally occurs in semiconductors when the energy of incident photons matches the bandgap energy. This allows the material to absorb photons, exciting electrons from the valence band (VB) to the conduction band (CB) and leaving behind holes in the VB, thus forming electron‐hole pairs. These excited electrons relax to lower energy states by either radiative relaxation (emitting photons) or non‐radiative relaxation (generating phonons, i.e., heat).^[^ ^41^ ^]^ When energy is released as phonons, it leads to local lattice heating, with the resulting temperature distribution depending on the material's optical absorption and recombination properties (Figure 2b2).^[^ ^42^ ^]^

The thermal vibration of molecules typically occurs in carbon‐based nanomaterials, such as graphene and carbon nanotubes. These materials are distinguished by their abundance of π─bonds, which have relatively weak binding energies. As a result, only a small amount of energy is required to excite electrons from z‐orbitals to π*‐orbitals. When the energy of the incident photons matches the energy of the intramolecular electronic transitions, the absorbed photons promote electrons from the highest occupied molecular orbital (HOMO) to the lowest unoccupied molecular orbital (LUMO).^[^ ^43^ ^]^ Notably, carbon materials, with their extensive network of conjugated π‐bonds, can absorb photons across the solar spectrum, leading to a series of π–π* transitions.^[^ ^35^, ^38^ ^]^ Following these transitions, excited electrons relax back to the ground state via electron‐phonon coupling, releasing vibrational energy (phonons) in the process and increasing the macroscopic temperature of the material (Figure 2c2).

## 4 De‐Icing Performance Based on Three Mechanisms

Building upon the three described primary photothermal mechanisms, materials such as metals, ceramics, semiconductors, and carbon‐based substances have been developed for de‐icing applications. This section highlights recent advancements in utilizing these materials for effective de‐icing, providing a comprehensive overview of the underlying mechanisms. Additionally, it examines the advantages and limitations of each approach in practical applications, offering insights into their potential and challenges in real‐world scenarios.

### 4.1 De‐Icing Performance of Plasmonic Localized Heating

Metallic materials, such as Au,^[^ ^44^ ^]^ Ag,^[^ ^45^ ^]^ and Pd^[^ ^46^ ^]^ nanoparticles, are excellent candidates to achieve photothermal effect due to their strong plasmonic responses within the solar spectrum. When exposed to solar irradiation, photons can become trapped within these metallic nanostructures, inducing the LSPR effect. The fabrication of these metallic nanomaterials uses methods involving galvanic replacement reaction,^[^ ^47^ ^]^ displacement deposition,^[^ ^48^ ^]^ spray coating,^[^ ^48^ ^]^ sputter deposition,^[^ ^49^ ^]^ laser surface direct writing,^[^ ^50^ ^]^ sol‐gel,^[^ ^51^ ^]^ nano masking,^[^ ^52^ ^]^ self‐assembly,^[^ ^44^ ^]^ redox reaction,^[^ ^45^ ^]^ electroplating,^[^ ^32^ ^]^ or a combination of these methods.^[^ ^45^ ^]^ These versatile fabrication approaches enable precise control over the structure and properties of metallic nanomaterials.

**Figure 3:**
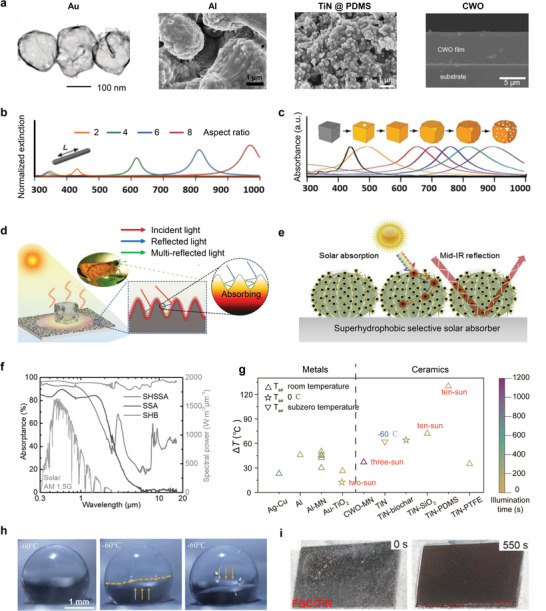
De‐icing performance based on plasmonic localized heating. a) TEM and SEM images of typical nanoparticles that exhibit plasmonic localized heating. Reproduced with permission.^[^ ^45^, ^50^, ^53^ ^]^ Copyright 2016, American Chemical Society; Copyright 2022, Elsevier; Copyright 2022, Wiley‐VCH GmbH; Copyright 2021, Cell Press. b) The variation of spectra as a function of nanorod aspect ratio of nanorods. Adapted with permission.^[^ ^57^ ^]^ Copyright 2014, Springer Nature. c) UV‐visible absorbance spectra of Ag nanocubes and Au nanocages. The Au nanocages are prepared by adding different volumes of 0.1 mM HauCl_4_ solution: from left to right on the x‐axis, 0, 0.3, 0.5, 1.0, 1.5, 2.0, 4.0, and 5.5 mL. Adapted with permission.^[^ ^58^ ^]^ Copyright 2014, Springer. d) Month eye‐inspired design for sunlight trapping. Adapted with permission.^[^ ^50^ ^]^ Copyright 2021, Elsevier. e) Cactus‐inspired design for sunlight trapping and the f) overall spectral absorptance across the UV, visible, and infrared wavelengths on the surface of a superhydrophobic selective solar absorber (SHSSA), superhydrophobic black absorber (SHB), and selective solar absorber (SSA). Adapted with permission.^[^ ^59^ ^]^ Copyright 2021, Cell Press. g) Temperature rise of different photothermal designs under different substrate temperature and sunlight illuminations. Data obtained from literature sources.^[^ ^33^, ^48^, ^49^, ^50^, ^51^, ^53^, ^59^, ^60^ ^]^ For a Summary of the data, please see Table S2 (Supporting Information). h) Photograph of a sessile water droplet residing on a surface which is at ultralow cryogenic temperatures down to ‐60 °C before freezing on a TiN‐particle decorated superhydrophobic surface. Reproduced with permission.^[^ ^59^ ^]^ Copyright 2021, Cell Press. i) Complete de‐icing achieved on an FBC/TiN nanoparticle‐coated surface under sun illumination. Reproduced with permission.^[^ ^60^ ^]^ Copyright 2022, Elsevier.

Among metallic materials, Au is the most extensively studied owing to its excellent light‐to‐heat conversion efficiency and its versatility in tailoring its size and structure. This adaptability enables the formation of various nanostructures, such as nanoparticles, nanorods, and hollow spheres (**Figure** **3**a).^[^ ^45^, ^50^, ^53^ ^]^ Notably, Au not only exhibits desirable photothermal properties independently but can also be hybridized with other materials to enhance photothermal efficiency. Examples include gold‐graphene oxide and gold‐titanium dioxide composites.^[^ ^54^ ^]^ Apart from noble metals like Au, more cost‐effective metallic nanomaterials, such as Cu^[^ ^55^ ^]^ and Al,^[^ ^56^ ^]^ also demonstrate remarkable solar absorbance exceeding 96%. Beyond metallic materials, certain ceramics, such as TiN^[^ ^53^ ^]^ and CWO,^[^ ^53^ ^]^ also manifest the plasmonic localized heating effect. For instance, CWO nanoparticles can realize efficient near‐infrared (NIR) absorption through LSPR. When combined with benzotriazole nanoparticles, these materials achieve near‐perfect spectral selectivity, with benzotriazole absorbing ultraviolet (UV) light and CWO absorbing NIR photons, resulting in a combined UV and NIR absorption exceeding 90%.^[^ ^53^ ^]^

The geometric design of nanoparticles plays a critical role in modulating their optical absorption properties. Adjusting the size and aspect ratio of metallic nanoparticles can shift the LSPR band position (Figure 3b).^[^ ^57^ ^]^ Another approach involves the creation of hollow nanostructures (Figure 3c) or the reduction of shape symmetry, which can broaden the LSPR spectral band and enable enhanced absorption over a wider range.^[^ ^58^ ^]^ Beyond nanoscale engineering, microscale structuring also plays a key role in affecting photothermal effect. When combining nanomaterials, microstructures can form numerous photothermal traps to concentrate solar energy because of the multiple reflections of the light within the trap. Such designs could also be found in nature. For example, the structure of the moth eye exhibits a black coloration, which serves to minimize light reflection within the eye and thereby avoid natural predators. This coloration results from the specific microscale structures present in the moth‐eye (Figure 3d).^[^ ^50^ ^]^ Another example is the cactus with hierarchical architecture (Figure 3e).^[^ ^59^ ^]^ Combining such nature‐inspired structural design with ceramic plasmonic titanium nitride (TiN) nanoparticles allows for a high solar absorbance (≈90%) (Figure 3f) and low IR absorption/emission (≈42%) over the mid‐IR range (>2.5 mm).^[^ ^59^ ^]^ These properties lead to a significant temperature rise (61 °C) under one‐sun illumination, outperforming blackbody‐like surfaces (52 °C).

Despite numerous configurations of photothermal surface designs, quantifying their photothermal efficacy is relatively simple, which usually necessitates the measurement of surface temperature rise during exposure to sun light. Figure 3g summarizes reported temperature rises for various photothermal materials involving metals and ceramics, with data spanning substrate temperatures from 15 to 70 °C under one‐sun illumination. Table S2 (Supporting Information) contains the full dataset used in the analysis with all sources and methods.^[^ ^33^, ^48^, ^49^, ^50^, ^51^, ^53^, ^59^, ^60^ ^]^ Note that while certain studies have documented comparatively high temperature rises, these were achieved under concentrated solar irradiation, such as ten‐sun illumination.^[^ ^53^ ^]^ Leveraging the inherent advantages in surface heating, a multitude of surfaces have demonstrated efficient anti‐icing and de‐icing functionalities. For instance, a TiN particle‐decorated superhydrophobic surface allows sessile water droplets to resist freezing at ultralow cryogenic temperatures down to −60 °C (Figure 3h),^[^ ^59^ ^]^ far below the operational requirements of most applications. Furthermore, sunlight‐mediated heating of FBC/TiN nanoparticle‐coated surfaces facilitates the complete removal of accreted ice (Figure 3i),^[^ ^60^ ^]^ showcasing high de‐icing efficacy and significant potential for practical applications.

### 4.2 De‐Icing Performance of Materials Undergoing Non‐Radiative Relaxation

**Figure 4:**
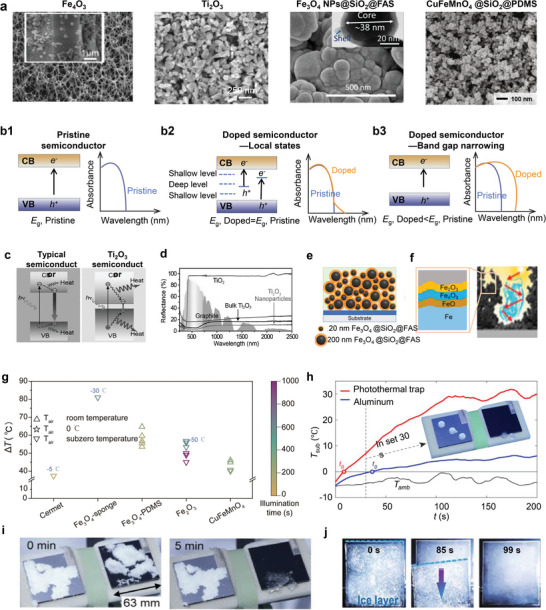
De‐icing performance of materials undergoing non‐radiative relaxation. a) SEM images of representative semiconductor particles and composites. Reproduced with permission.^[^ ^41^, ^61^ ^]^ Copyright 2017, Wiley‐VCH GmbH; Copyright 2023, American Chemical Society; Copyright 2022, Elsevier; Copyright 2021, Elsevier. b) The band structure and the correspondent optical absorption curves of a pristine semiconductor, doping‐induced shallow‐level and deep‐level states, and doping‐induced bandgap narrowing. Adapted with permission.^[^ ^67^ ^]^ Copyright 2015, the Royal Society of Chemistry. c) Schematics of electro‐hole generation and relaxation enabled by the conventional bulk Ti_2_O_3_ with a comparatively high bandgap and Ti_2_O_3_ nanoparticles with a narrow bandgap (0.1 eV), and d) the correspondent reflectance spectra of these two materials as well as commercial TiO_2_ and graphite. Reproduced with permission.^[^ ^41^ ^]^ Copyright 2017, Wiley‐VCH GmbH. e) Light trapping effect due to the hierarchical structure, together with the non‐radiative relation effect of Fe_3_O_4_ nanoparticles, resulting in efficient surface heating. Reproduced with permission.^[^ ^61^ ^]^ Copyright 2022, Elsevier. f) Photothermal design using hierarchical structure as well as varied surface compositions Reproduced with permission.^[^ ^64^ ^]^ Copyright 2021, National Academy of Sciences. g) Temperature rise of non‐radiative relaxation designs under different substrate temperature and sunlight illuminations in literature. Data obtained from literature sources.^[^ ^21^, ^61^, ^64^ ^]^ For a Summary of the data, please see Table S3 (Supporting Information). PDMS is the abbreviation of Polydimethylsiloxane. h) The efficiency in photothermal trap of cermet (MT1300 mirotherm®, Alanod GmbH) coating leads to a substantial rise in surface temperature under a subzero ambient temperature, which promotes the melting and complete shedding of frozen droplets and i) snow after 5 min of illumination. Adapted under the terms of the CC‐BY Creative Commons Attribution 4.0 International license.^[^ ^21^ ^]^ Copyright 2022, The Authors, some rights reserved, exclusive licensee AAAS. j) The efficient de‐icing performance manifested on a double‐layer solar thermal coating composed of SiO_2_ nanospheres and CuFeMnO_4_ semiconductor. Reproduced with permission.^[^ ^61^ ^]^ Copyright 2021, Elsevier.

Non‐radiative relaxation normally manifest in semiconductors, particularly those involving metal oxides (*e.g*., Fe_3_O_4_, Ti_2_O_3_, MnFe_2_O_4_, and NiO) and chalcogenides (*e.g*., Cu_7_S_4_ and Cu_12_Sb_4_S_13_), as shown in **Figure** **4**a.^[^ ^41^, ^61^ ^]^ The fabrication of these materials can rely on techniques involving milling,^[^ ^41^ ^]^ spraying,^[^ ^61^ ^]^ deposition,^[^ ^61^ ^]^ calcination,^[^ ^62^ ^]^ solid‐state reaction,^[^ ^63^ ^]^ pulsed laser deposition and oxidization,^[^ ^64^ ^]^ dopant incorporation.^[^ ^62^, ^65^ ^]^ Among those, doping has attracted tremendous attention owing to its ability to modify the bandgap of semiconductors. Normally, a narrower bandgap allows semiconductors to absorb a broader range of wavelengths. Immense research has focused on utilizing doping to achieve this. One key objective is to introduce shallow and deep‐level states, which serve as centers for optical excitation and relaxation. This process adds a “shoulder” to the absorbance curve of the doped semiconductor compared to its pristine counterpart, effectively expanding the spectral range (Figure 4b1–b2). A typical example is the creation of oxygen‐deficient MoO_3_‐x quantum dots, which exhibit broad light absorption spanning the UV, visible, and near‐IR regions. This broadening is attributed to oxygen vacancy‐induced electronic transitions between defect energy levels and other states, rather than localized surface plasmon resonance effects.^[^ ^66^ ^]^

Parallel to the introduction of local state inside the bandgap, other doping approaches can directly narrow the bandgap by shifting the conduction or valence band positions (Figure 4b3).^[^ ^67^ ^]^ One notable example is the intrinsically large bandgap (≈3 eV) of TiO₂ limits its response to wavelengths greater than 400 nm. Bandgap narrowing efforts have included developing disorder‐engineered TiO₂ nanocrystals,^[^ ^65^ ^]^ ordered mesoporous black TiO_2_,^[^ ^68^ ^]^ and TiO_2_ nanotubes.^[^ ^69^ ^]^ While these approaches have reduced the bandgap to ≈1.5 eV, achieving full‐spectrum solar absorption requires an even narrower bandgap (<0.5 eV). Alternatively, Ti_2_O_3_ nanoparticles exhibit an ultranarrow bandgap (<0.1 eV), a value significantly less than most photons from solar irradiation. This property enables the generation of above‐bandgap electron‐hole pairs under sunlight irradiation and the later relaxation of the electrons to the band edges, leading to the conversion of the extra energy into heat through a thermalization process (Figure 4c).^[^ ^41^ ^]^ With enhanced light scattering and minimal reflectance (<10%) (Figure 4d),^[^ ^61^ ^]^ Ti_2_O_3_ nanoparticles efficiently absorb solar energy across the spectrum.

Beyond intrinsic properties, incorporating hierarchical structures into surface designs can enhance light trapping. Examples can be found in the utilization of Fe_3_O_4_ nanoparticles having two distinct sizes. This design presents both light trapping and non‐radiative heating effects, synergistically boosting surface heating (Figure 4e).^[^ ^64^ ^]^ In addition to the single composite used in hierarchical structure design, using multiple composites that combine different photothermal effects has also been adopted. As exemplified by a hierarchical photothermal coating, the existence of three different iron oxide layers not only boosts non‐radiative relaxation, but also facilitates the formation of microstructures that enable light trapping and extended light trajectories, leading to superior anti‐reflection performance and high solar‐to‐heat efficiency (Figure 4f).^[^ ^64^ ^]^

Figure 4g summarizes the temperature rises achieved by semiconductor‐based solar heating materials. The full dataset, including sources and methods, is provided in Table S3 (Supporting Information).^[^ ^21^, ^61^, ^64^ ^]^ Most designs show a temperature rise between 40 and 60 °Cunder one‐sun illumination, with lower rises in subzero conditions compared to room temperature. Notably, a sponge‐like structure decorated with Fe_3_O_4_ nanoparticles achieved a significant temperature rise of 80 °C, even at −30 °C. This performance is attributed to the high light‐trapping efficiency of the micro‐ holes on the sponge‐like surface.^[^ ^21^ ^]^ Leveraging the inherent advantages of non‐radiative relaxation‐driven surface heating, numerous studies have showcased the efficacy of these materials in de‐icing, involving shedding of melted droplets (Figure 4h),^[^ ^21^ ^]^ clearing snow (Figure 4i),^[^ ^21^ ^]^ and removing bulk ice (Figure 4j)^[^ ^61^ ^]^ under solar irradiation.

### 4.3 De‐Icing Performance of Materials Undergoing Thermal Vibration

**Figure 5:**
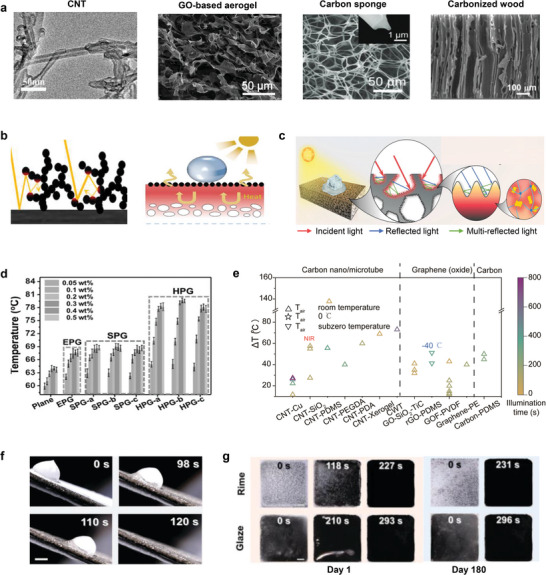
De‐icing performance of materials undergoing thermal vibration in molecules. a) SEM images of representative carbon‐based particles and composites. Reproduced with permission.^[^ ^70^, ^72^, ^74^ ^]^ Copyright 2022, Elsevier; Copyright 2018, Wiley‐VCH GmbH; Copyright 2017, Wiley‐VCH GmbH; Copyright 2019, American Chemical Society. b) The structures formed by carbon nanospheres exhibit the enhanced light trapping ability, while the underlying porous PDMS layer shows the capability to insulate conduction losses into the substrate, both of which govern the interface temperature rise. Reproduced with permission.^[^ ^73^ ^]^ Copyright 2023, Elsevier. c) Hierarchical rGO structures showing the increased light trapping. Adapted with permission.^[^ ^76^ ^]^ Copyright 2020, Royal Society of Chemistry. d) Temperature variation of different samples with various rGO contents under one‐sun illumination for 400 s. EPG, SPG, and HPG refers to emulsion templated PDMS/rGO, sugar templated PDMS/rGO, and hierarchical structured PDMS/rGO, respectively. Reproduced with permission.^[^ ^76^ ^]^ Copyright 2020, Royal Society of Chemistry. e) Temperature rise of molecular thermal vibration designs under different ambient temperatures and sunlight illuminations. Data obtained from literature sources.^[^ ^48^, ^71^, ^73^, ^75^, ^76^, ^77^, ^78^, ^79^, ^82^ ^]^ For a Summary of the data, please see Table S4 (Supporting Information). f) Time‐lapse images of frozen droplet sliding on the hierarchical structured PDMS/rGO coating, at −35 °C ambient temperature under one‐sun illumination. Reproduced with permission.^[^ ^76^ ^]^ Copyright 2020, Royal Society of Chemistry. g) Long‐term solar thermal deicing performance under rime and glaze icing conditions. Reproduced with permission.^[^ ^76^ ^]^ Copyright 2020, Royal Society of Chemistry.

While materials featuring mechanisms of plasmonic localized heating and non‐radiative relaxation exhibit wavelength‐specific light absorption, carbon‐based materials that induce molecular thermal vibration possess the unique capability to absorb light across the whole solar spectrum. In addition to this unique capability, they offer several advantages, including low thermal radiation, good biocompatibility, low toxicity, and abundant availability.^[^ ^35^ ^]^ Commonly explored carbon‐based materials include carbon nanotubes (**Figure** **5**a),^[^ ^48^, ^70^ ^]^ graphene,^[^ ^71^ ^]^ graphene oxide (Figure 5a),^[^ ^72^ ^]^ carbon microspheres,^[^ ^73^ ^]^ carbon sponge (Figure 5a)^[^ ^74^ ^]^ and ink,^[^ ^75^ ^]^, and carbonized wood.^[^ ^74^ ^]^ A significant challenge with carbon materials is their poor adhesion and mechanical stability on substrates. To address this, fabrication technologies primarily relies on the combination of polymeric solvents such as PDMS,^[^ ^71^, ^73^, ^76^ ^]^ polyvinylidene fluoride (PVDF),^[^ ^70^, ^77^ ^]^ polyethylene (PE),^[^ ^71^ ^]^ poly(ethylene glycol) diacrylate (PEGDA),^[^ ^70^ ^]^ polyurethane (PU),^[^ ^78^ ^]^ and modified silicone resin.^[^ ^79^ ^]^ However, these carbon materials exhibit an inherent tendency to agglomerate when dispersed in solvents. To address this challenge, the incorporation of additional materials, such as SiO_2_ nanoparticles,^[^ ^70^ ^]^ has been employed to enhance dispersion.

To further amplify the photothermal effect, some studies have explored the combination of carbon materials with other nanoparticles exhibiting complementary photothermal effects, such as TiC.^[^ ^79^ ^]^ Beyond flat substrates, carbon nanomaterials can be deposited on pre‐structured surfaces, such as xerogels^[^ ^80^ ^]^ or patterned PE substrates.^[^ ^71^ ^]^ These microstructured substrates leverage light‐trapping effects to enhance the photothermal response.^[^ ^76^ ^]^ Interestingly, carbonized natural materials (Figure 5a),^[^ ^81^ ^]^ such as plants and wood, offer a sustainable and efficient approach to photothermal de‐icing, due to their high solar thermal efficiency and low thermal convection loss.

Aside from material selection, structural configuration plays a critical role in optimizing performance. For example, highly disordered nanosized voids and cavities in carbon spheres, with dimensions smaller than sunlight wavelengths, enhance light trapping. When combined with insulating layers like porous PDMS, this design confines heat at the interface, increasing interfacial temperature (Figure 5b).^[^ ^73^ ^]^ As a step forward, hierarchical structure designs represent another avenue for improvement. A remarkable of this example is a coating of reduced graphene oxide (rGO) and PDMS featuring micro hole and nano wrinkles, with dimensions aligned to the solar spectrum (300–2500 nm), achieves multiple internal light reflections and absorption rates exceeding 96% (Figure 5c).^[^ ^76^ ^]^ Furthermore, the photothermal properties of these materials can be further modulated by tuning the rGO content, wherein a 0.4 wt% increase in the content can lead to an 8 °C rise in the surface temperature (Figure 5d).^[^ ^76^ ^]^

Figure 5e summarizes the temperature rises reported in literature using nanomaterials for photothermal de‐icing.^[^ ^48^, ^71^, ^73^, ^75^, ^76^, ^77^, ^78^, ^79^, ^80^, ^82^ ^]^ The data, detailed in Table S4 (Supporting Information), reveal significant variability in performance, with temperature increases ranging from below 20 °C to over 70 °C under one‐sun illumination.^[^ ^48^, ^71^, ^73^, ^76^, ^77^, ^78^, ^79^ ^]^ While some studies report extreme rises exceeding 150 °C, these results were achieved under concentrated solar irradiation (e.g., twenty five suns), which is less relevant to standard one‐sun conditions. Notably, GO‐SiO₂ composites under five‐sun light intensity exhibited lower temperature rises than many other carbon materials tested under one‐sun conditions, highlighting the importance of careful material and formulation selection. It's noteworthy that most of the reported measurements were conducted at room temperatures, limiting their direct applicability to subzero environments typical of real‐world icing scenarios. Nevertheless, carbon‐based materials have demonstrated effective de‐icing capabilities, shedding frozen droplets (Figure 5f),^[^ ^76^ ^]^ frost, bulk glaze, and rime ice (Figure 5g).^[^ ^76^ ^]^ Through appropriate surface engineering, such de‐icing ability has been shown to persist for up to 180 days, underscoring the long‐term potential of these materials.

Together, the above discussion highlights the shared strengths of different photothermal materials in global surface heating response and de‐icing performance, despite their varying mechanisms. **Table** **1** provides a comparison of the three photothermal mechanisms regarding their photothermal absorbance, de‐icing performance, pros and cons for de‐icing, and scope of use, with detailed supplementary information in Table S5 (Supporting Information). These insights offer valuable guidance for future research and the development of advanced photothermal materials tailored for practical de‐icing applications.

**Table 1:** Comparison of three photothermal mechanisms for efficient de‐icing.

	Plasmonic Localized Heating	Non‐Radiative Relaxation	Thermal Vibration in Molecules
Solar absorbance	>88%, mostly larger than 90%	>80%, mostly larger than 90%	>60%, mostly larger than 90%
De‐icing performance	Under one‐sun illumination, necessitate 64–600 s; Under illumination intensity larger than one‐sun, necessitate 3–358 s	Under one‐sun illumination, necessitate 790 s; Under illumination intensity less than one sun, necessitate 12 min; Under illumination intensity larger than one sun, necessitate 50 s‐10 min	Under one‐sun illumination, necessitate 59 s‐15 min; Under illumination intensity less than one sun, necessitate 26 min; Under illumination intensity larger than one sun, necessitate 5–358 s
Pros	Tunable heating; Localized heating; Fast response in temperature change; High photothermal conversion efficiency	Wide range of material options; Strong extinction coefficients in the NIR region	Broad bandwidth; Wide range of material options; High stability in heat generation; High chemical stability under ambient conditions
Cons	Narrow bandwidth; Limited material choice; Environmental sensitivity; Potential for local overheating	Relatively narrow bandwidth; Slow response speed in non‐radiative relaxation for some materials; Sensitivity to defects and impurities within materials	Relatively low thermal vibration efficiency; Possible oxidation or decomposition in high‐temperature or strongly oxidative environments
Scope of use	Ideal for transparent or translucent devices requiring a high transmission in the visible range, such as windshields, eyeglasses, and windows	Ideal for applications requiring efficient heating and rapid temperature increase, such as solar panels, aircraft, and architectural glass	Ideal for low‐power applications requiring precise temperature control, such as solar water heater, precision equipment, and small‐scale photothermal power generation device
References	[33, 48, 49, 50, 51, 53, 59, 60, 83]	[21, 61, 64]	[48, 71, 73, 75, 76, 78, 79, 80, 82]

## 5 De‐Icing Performance Via the Combination of Photothermal Effect with Other Surface Designs

Despite advancements in photothermal surface designs for efficient de‐icing, no single design can fully address the wide range of challenges encountered across varying temporal and spatial scales under complex meteorological conditions. In this section, we discuss recent progress in photothermal design in integrating photothermal designs with complementary surface engineering approaches. These include superhydrophobic, slippery surface properties, and use of phase‐change materials, and electro‐thermal methods. To better evaluate their de‐icing performance, we categorize these approaches based on two key characteristics: efficient meltwater removal and all‐weather de‐icing capabilities.

### 5.1 Efficient Meltwater Removal During De‐Icing

As de‐icing on photothermal surfaces is normally achieved by ice transport via the interfacial meltwater, the retention of meltwater is a significant challenge for many photothermal surfaces, especially those with hydrophilicity properties. This is because the retained meltwater can refreeze under cold, sunlight‐absent conditions, exacerbating icing or frosting. To facilitate complete shedding of meltwater, water‐repellent photothermal surfaces with superhydrophobic or slippery properties have been developed. This section explores recent advancements in combining photothermal designs with these properties to facilitate efficient de‐icing while mitigating meltwater retention.

#### 5.1.1 De‐Icing Via the Combination of Photothermal and Superhydrophobic Properties

**Figure 6:**
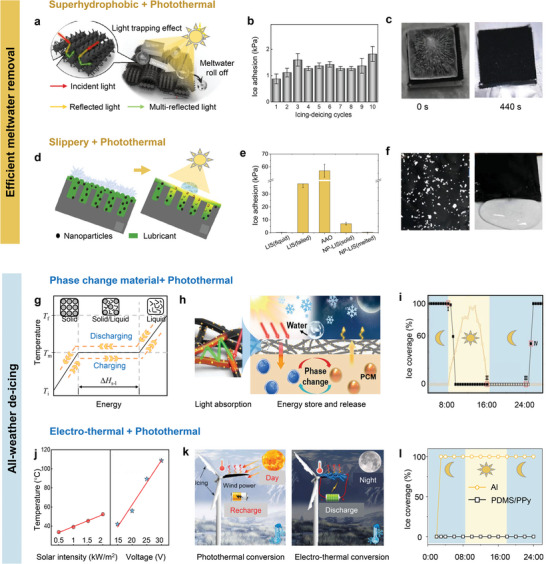
Efficient de‐icing via the combination of photothermal effect with other surface designs. a) Design of superhydrophobic surface with photothermal effect showing ability in light trapping and complete meltwater removal. Adapted with permission.^[^ ^32^ ^]^ Copyright 2021, American Chemical Society. b) Ultra‐low interfacial ice adhesion. Adapted with permission.^[^ ^86^ ^]^ Copyright 2024, Royal Society of Chemistry. c) Complete de‐icing performance. Reproduced with permission.^[^ ^32^ ^]^ Copyright 2021, American Chemical Society. d) Slippery surface with photothermal effect promotes complete meltwater removal. Adapted with permission.^[^ ^92^ ^]^ Copyright 2020, Elsevier. e) Significant reduction in ice adhesion on slippery photothermal surface upon sunlight illumination. Adapted with permission.^[^ ^92^ ^]^ Copyright 2020, Elsevier. f) Complete meltwater removal. Reproduced with permission.^[^ ^95^ ^]^ Copyright 2021, Springer. g) The working principle of solid‐liquid phase change materials. Adapted with permission.^[^ ^100^ ^]^ Copyright 2022, Elsevier. h) Schematic of all‐weather de‐icing jointly achieved by phase change materials with photothermal effect^[^ ^102^ ^]^ and the associated i) all‐weather de‐icing performance. Adapted with permission.^[^ ^102^ ^]^ Copyright 2022, Wiley‐VCH GmbH. j) Photothermal and electrothermal response. Adapted with permission.^[^ ^106^ ^]^ Copyright 2024, Elsevier. k) Schematic of all‐weather de‐icing jointly achieved by photothermal and electrothermal design. Adapted with permission.^[^ ^106^ ^]^ Copyright 2024, Elsevier. l) All‐weather de‐icing performance. Adapted with permission.^[^ ^21^ ^]^ Copyright 2021, Wiley‐VCH GmbH.

Superhydrophobic surfaces, characterized by low surface energy and reduced liquid‐solid contact area, offer an effective approach for manipulating interfacial water and ice.^[^ ^84^ ^]^ The corporation of superhydrophobicity with photothermal effects has recently demonstrated efficiency in promoting ice melting and meltwater shedding. As the key to achieving extreme water repellency of superhydrophobic surfaces necessitates the integration of micro‐ and nanostructures,^[^ ^32^, ^85^ ^]^ current photothermal superhydrophobic surface designs have focused on decorating photothermal nanomaterials on engineered microstructures. This approach also harnesses the advantage of efficient light trapping via microstructures. For example, photothermal surfaces with carbon frameworks achieve efficient heating and complete meltwater shedding due to their hierarchical structure and low surface energy, leaving a clean, dry surface (**Figure** **6**a).^[^ ^32^ ^]^ Similar designs can render ultralow ice adhesion (as low as 2 kPa), which is maintained after repeated icing‐deicing cycles (Figure 6b).^[^ ^86^ ^]^ This enables the rapid removal of ice and meltwater (Figure 6c).^[^ ^32^ ^]^

Despite notable progress in such designs for efficient de‐icing, challenges remain during melting/defrosting cycles. Superhydrophobic surfaces may suffer from structure damage or chemical degradation of the top hydrophobic layer by shear forces induced during repetitive melting/defrosting cycles.^[^ ^87^ ^]^ A previous study demonstrated long‐term cyclic/repetitive freezing‐melting experiments on superhydrophobic surfaces, performing ≈1000 cycles, equivalent to six months of continual testing.^[^ ^15^ ^]^ These experiments were conducted on a CuO‐silane‐based superhydrophobic surface, which are typically fragile. While no mechanical damage was observed on the surfaces even after six months of testing, detailed characterization revealed some performance degradation, likely due to hydrolysis of the coating on the superhydrophobic surfaces.^[^ ^15^ ^]^ Moreover, under a humid and low‐temperature environment, hierarchical textures or the hydrophobic coating of superhydrophobic surfaces become susceptible to the Cassie‐to‐Wenzel transition^[^ ^88^ ^]^ or penetration of condensed droplets,^[^ ^89^ ^]^ leading to a compromised superhydrophobic behavior that presents ice‐sticky properties.^[^ ^90^ ^]^ The resulting full coverage of the ice inevitably leads to a sacrifice in light‐trapping efficiency. Potential solutions include structurally homogeneous nanostructures to prevent droplet penetration and self‐refreshing mechanisms through coalescence‐induced droplet shedding. Such enhancements have demonstrated ice‐free performance at ambient temperatures as low as ‐50 °C, making them promising for extreme conditions.^[^ ^64^ ^]^

#### 5.1.2 De‐Icing Via the Combination of Photothermal and Slippery Properties

Unlike superhydrophobic surfaces, slippery surfaces reduce ice adhesion by forming a molecularly smooth liquid‐solid interface, enabled by a water‐immiscible lubricant.^[^ ^23^, ^91^ ^]^ Such a smoothness is endowed by the presence of a water‐immiscible lubricant, which can significantly eliminate the pinning of water. Combining photothermal effects with slippery surface properties has shown advantages in rapid meltwater and ice removal under light irradiation (Figure 6d).^[^ ^92^ ^]^ The success of this approach relies on selecting appropriate lubricants (*e.g*., silicone oil,^[^ ^93^ ^]^ cocoa oil,^[^ ^92^ ^]^ poly(methyhydrosiloxane)^[^ ^94^ ^]^) and photothermal materials (*e.g*., Fe_3_O_4_ nanoparticles,^[^ ^92^, ^93^, ^95^ ^]^ multiwalled carbon nanotubes,^[^ ^94^ ^]^ Cu(OH)_2_ nanowires,^[^ ^94^ ^]^ candle shoot,^[^ ^95^ ^]^ or graphene oxide^[^ ^96^ ^]^). For example, incorporating Fe_3_O_4_ nanoparticles into mineral oil enhances interfacial heating during solar irradiation or near‐IR laser irradiation, significantly lowering ice adhesion compared to surfaces with only slippery lubricants or photothermal materials (Figure 6e).^[^ ^92^ ^]^ This dual‐function design allows complete ice shedding, leaving a dry surface (Figure 6f).^[^ ^95^ ^]^

### 5.2 All‐Weather De‐Icing

While photothermal designs with superhydrophobic or slippery properties excel in meltwater removal, their efficiency diminishes in low‐light conditions (*e.g*., at night or on cloudy days). Ice formation under real‐world conditions can vary significantly in thickness, texture, and size, affecting light transmission and de‐icing efficiency. Clear, bubble‐free ice exhibits minimal absorption between 180 and 700 nm, increasing only in the IR region.^[^ ^97^ ^]^ On this basis, materials like carbon nanomaterials, with broad‐spectrum light absorption, are preferred for melting clear ice.^[^ ^35^ ^]^ However, for impure or bubble‐laden ice, light transmittance decreases, reducing the effectiveness of all photothermal materials.^[^ ^97^ ^]^ Thus, there is a pressing need to develop technologies that can solve this intermittent availability and supply‐demand discordance at night or on cloudy days. Currently, meeting the demand to achieve all‐weather de‐icing can build on two main technologies that can generate heat at night or on cloudy days. One is the employment of phase‐change materials (PCMs) that can store and release heat in an on‐demand manner, while the other is using the electro‐thermal approach.

#### 5.2.1 De‐Icing Via the Combination of Photothermal Effect and Phase Change Materials

The ability to store, release, and control heat makes PCMs a promising candidate for bridging energy supply‐demand mismatches.^[^ ^98^ ^]^ Among three types of phase transformations of PCMs, solid‐liquid transitions have recently attracted significant attention owing to their low volume change, high thermal storage capacity, and constant phase‐change temperature.^[^ ^99^ ^]^ The working mechanism involves heat absorption, phase change, and heat release in a continuous cycle. The entire process is referred to as charging, while the reverse process is called discharging (Figure 6g).^[^ ^100^ ^]^

Building upon this mechanism and method, PCMs with photothermal effects can continuously heat the surface under low‐density sunlight or at night, serving as a complementary solution to photothermal materials which only function effectively during daytime.^[^ ^101^ ^]^ One remarkable example is a biomimetic design inspired by polar bears that consists of a fat layer serving as the energy storage and release component, a black skin layer for solar absorption and conversion, and a tubular hair layer that ensures self‐cleaning functionality. By adopting SiO_2_‐encapsulated n‐tetradecane as the phase change materials, Ox‐MWCNTs as the photothermal layer, and hexamethyldisilazane as the superhydrophobic layer (Figure 6h), the design achieves >96% solar absorbance, a temperature rise of 40 °C, and superior anti‐icing and de‐icing performance throughout the day (Figure 6i).^[^ ^102^ ^]^ However, PCM‐based systems face challenges in achieving high encapsulation rates, thermal conductivity, and photothermal conversion efficiency simultaneously.^[^ ^103^ ^]^ One possible solution is the adoption of a dual‐ shell design, such as Cu_2_O and 3D Cu_2‐x_S encapsulating phase change materials (n‐Eicosane), which deliver exceptional solar absorption (>96%), high thermal storage (99.2%), and effective de‐icing even in low temperatures and high humidity.^[^ ^104^ ^]^

#### 5.2.2 De‐Icing Via the Combination of Photothermal Effect with Electro‐Thermal Approach

Unlike phase‐change materials (PCMs), which require preliminary heat storage for subsequent release, the electro‐thermal approach generates heat instantly, making it highly suitable for de‐icing applications, particularly for aircraft.^[^ ^1^, ^105^ ^]^ This method relies on the principle of converting electrical energy into thermal energy through electrical resistance. High‐resistance materials such as nichrome, tungsten, or carbon serve as heating elements that resist the flow of electrical current, dissipating energy as heat. As applied voltage increases, so does the surface temperature, demonstrating the efficiency of the electro‐thermal approach as a complementary de‐icing solution (Figure 6j).^[^ ^106^ ^]^ Despite its advantages, the electro‐thermal approach poses challenges. The required equipment and the significant energy loss during prolonged operation can create burdens, particularly in applications demanding lightweight systems and reduced energy consumption.

A hybrid approach combining photothermal and electro‐thermal properties offers a promising solution to these challenges. This integrated design alternates between photothermal and electro‐thermal functionalities based on sunlight intensity, minimizing energy input over a 24‐h cycle (Figure 6k).^[^ ^106^ ^]^ Such systems have demonstrated temperature rises of up to 75 °C under low‐voltage inputs and maintained ice‐free conditions even at extremely low ambient temperatures (−25 °C) during day‐night alternations^[^ ^22^ ^]^ or in cloudy weather (Figure 6l).^[^ ^21^ ^]^ Incorporating superhydrophobic properties into photothermal‐electrothermal designs further enhances de‐icing performance, which shows application potential especially in wind turbine blade systems.^[^ ^33^ ^]^ For instance, a composite surface with fluorinated epoxy resin and embedded PTFE nanoparticles as the hydrophobic component, carbon nanoparticles for sunlight absorption, and copper foil electrodes for electro‐thermal heating outperformed conventional electro‐thermal surfaces. This design achieved both light‐induced ice shedding and efficient electrothermal heating. With minimal electrical input (<0.4 W cm^−2^), it reduced ice adhesion to exceptionally low values (<5 kPa), enabling ice self‐shedding via gravity at adhesion thresholds as low as 20 kPa.^[^ ^12^, ^107^ ^]^

## 6 Conclusions and Outlook

Understanding the underlying photothermal physics holds great promise for achieving highly efficient de‐icing. Despite substantial progress made in this field, there is a pressing need to deepen our understanding of the mechanisms of photothermal conversion. This endeavor should be supported by the discovery of novel materials, innovations in ice detection methods, and the multidisciplinary integration of diverse scientific fields. Beyond exploring fundamental mechanisms, it is equally important to establish standardized approaches for evaluating photothermal and de‐icing performance. For photothermal performance assessment, while the measurement of solar absorbance using spectrophotometers is widely accepted, the evaluation of surface temperature rise has been conducted under diverse substrate temperatures, environmental humidity and temperatures, and even varying light intensities, all of which can influence the results. Moreover, the prevalent use of IR cameras for temperature measurement is prone to uncertainty due to improperly calibrated surface emissivity, ambient temperature, and humidity, as well as differences in measurement locations (ice layer or substrate), leading to potential deviations from the true values.

Similarly, assessing de‐icing performance presents challenges. The kinetics of ice melting are affected by multiple parameters, including the intensity and angle of incident light, the illuminated surface area, the size and thickness of ice blocks, the thickness and coating of the test surface, the temperature of the test surface, ambient temperature and humidity, and the insulation properties of the underlying support. The absence of a unified set of testing parameters across studies has significantly impeded the effective comparison and interpretation of photothermal de‐icing results in the literature.

**Figure 7:**
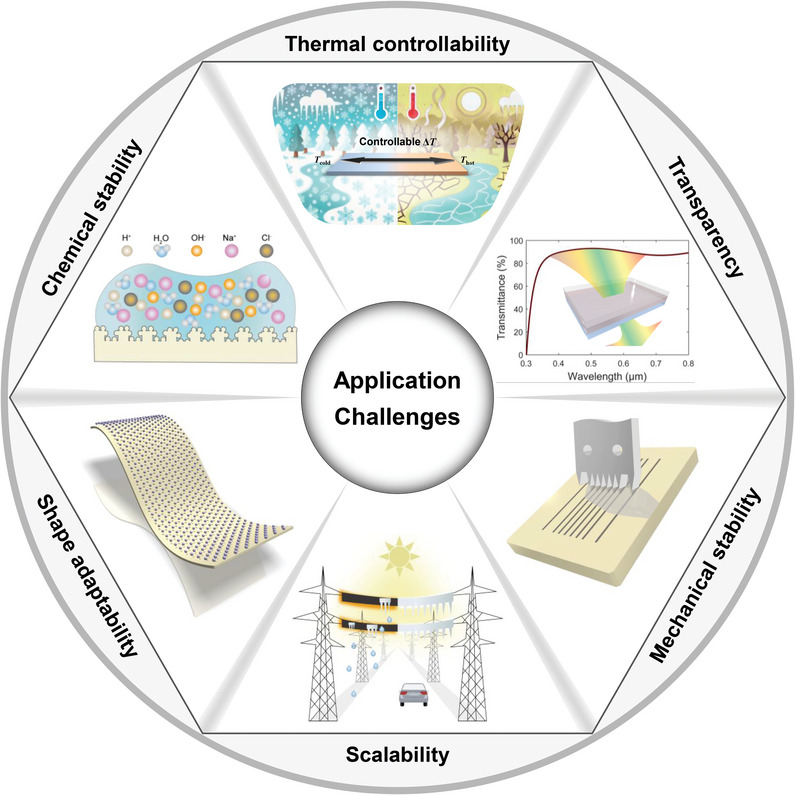
Schematic summarizing the application challenges to photothermal deicing. Challenges involve mechanical stability, chemical stability, thermal controllability, scalability, shape adaptability, and transparency.

Moving toward real‐world applications, six primary challenges emerge for photothermal de‐icing, as summarized in **Figure** **7**.

### 6.1 Mechanical Stability

The mechanical stability of photothermal materials poses a primary challenge, as many designs are made of nanomaterials which are prone to damage during operation. This challenge is especially pronounced in photothermal designs coupled with superhydrophobic or slippery properties, which often involve complex manufacturing processes^[^ ^90^, ^108^ ^]^ or liquid infusion^[^ ^109^ ^]^ that can suffer from mechanical wear or lubrication depletion.^[^ ^110^ ^]^ Recent strategies demonstrate that overcoming these challenges can rely on integrating photothermal materials with inherently hydrophobic polymers, such as PDMS^[^ ^85^ ^]^ and silicon resin,^[^ ^85^ ^]^ or optimizing the coating geometric and structural design, for instance, crafting micro architectures which act as a protective or sacrificial armor.^[^ ^111^ ^]^ By doing so, the coatings show strong resistance to mechanical abrasion, chemical corrosion, and thermal variation, all together contributing to durable de‐icing performance. Additionally, previous works on robust superhydrophobic surfaces with photothermal effect further support the potential for durable designs, showing resistance to water injection, sand impact, and even long‐term mechanical abrasion.^[^ ^84^, ^112^ ^]^ Similarly, the self‐repairing ability of slippery surfaces endows the surface with exceptional durability.^[^ ^113^ ^]^ However, challenges persist, including achieving thin coatings, scalability, and substrate compatibility. Optimized surface architectures and durable surface chemistry can alleviate these concerns. For example, micro‐thick, scalable coatings developed via physical vapor deposition^[^ ^114^ ^]^ or combined processes like salinization and impregnation^[^ ^84^ ^]^ have shown resilience to humid and cold environments for up to three years. Although their photothermal performance remains underexplored, these principles offer a foundation for future robust de‐icing solutions.

### 6.2 Thermal Controllability

While photothermal coatings are valuable for de‐icing, they can have adverse effects on surface temperature in certain climates. In cold, cloudy conditions, reduced heating effectiveness limits surface temperature rises, hampering de‐icing. Conversely, in hot climates, excessive heating can lead to system overheating, material stress, or cracking due to thermal expansion and contraction. Reported temperature rises for photothermal surfaces under one‐sun illumination typically range from 20 to 60 °C (Figures 3, 4, and 5e). However, some materials, such as Fe₃O₄ sponges, achieve rises to 80 °C, and CNT‐PDMS nanoparticles reach 135 °C under laser illumination.^[^ ^61^, ^70^ ^]^ Such overheating can strain systems, particularly in warm climates where surface temperatures need not exceed 0 °C for de‐icing. Furthermore, overheating can increase cooling demand in hot climates. Given that most outdoor industrial facilities operate above −40 °C and that ice melting necessitates only a surface temperature above 0 °C, it may not be necessary to use photothermal materials with ultra‐high performance. Thus, mitigating the adverse effects on surface temperature can resort to properly choosing the photothermal material that meet the practical thermal need.

### 6.3 Scalability

Achieving effective photothermal de‐icing capabilities at small scales is comparatively straightforward. However, application of photothermal coatings to large‐scale systems is fraught with significant challenges. This is because commercial viability of photothermal coatings is highly dependent on the fabrication techniques employed and the choice of materials used. The fabrication of photothermal surfaces should primarily consider the use of commercially available and economically viable products. To date, a substantial body of nanomaterials, particularly metallic nanoparticles such as Au and Ag, are prohibitively expensive for large‐scale deployment. Carbon‐based materials that meet the criteria may serve as a more suitable alternative. Nonetheless, imparting the required mechanical robustness to carbon materials necessitates the use of polymer binders, which increases the complexity of fabrication. Moreover, scaling up the fabrication process requires the innovative integration of multiple considerations, such as substrate compatibility, the design of the manufacturing process line, and the application of appropriate coating patterns. These challenges must be addressed to achieve the practical implementation of photothermal de‐icing coatings at a large scale.

### 6.4 Chemical Stability

In industrial environments, surfaces are often subjected to a variety of chemically corrosive and reactive substances, such as acidic and alkaline solutions, salt solutions, and organic solvents.^[^ ^115^ ^]^ Even in relatively benign outdoor environments, exposure to ultraviolet radiation, seawater, or acid rain, can be prevalent. If the surface coating lacks sufficient chemical stability, it can easily experience issues like peeling, cracking, or corrosion.^[^ ^116^ ^]^ This not only affects the aesthetic appearance of the surface but can also seriously undermine the service life and performance reliability of the equipment. One possible pathway is to rationally choose photothermal materials that show strong resistance to chemical and UV interactions or embed the photothermal materials into the chemically stable composites based on the correspondent working environments.^[^ ^85^ ^]^

### 6.5 Shape Adaptability

Industries increasingly require photothermal coatings for complex or irregular surfaces, such as those found in heat pumps,^[^ ^117^ ^]^ aircraft,^[^ ^118^ ^]^ and flexible solar cells,^[^ ^119^ ^]^ as they enable specialized functionalities, including expanded heat transfer area, enhanced lift, and improved energy harvesting efficiency. A key challenge in this context is the development of photothermal coatings which can be applied to and conform on the shape of these non‐planar or irregular‐shaped surfaces. A potential solution may lie in the judicious selection of photothermal materials with strong shape‐adaptability, coupled with the employment of intelligent and controllable coating fabrication techniques, such as spray coating, dip coating, or conformal chemical vapor deposition.

### 6.6 Transparency

Most photothermal designs exhibit a black appearance, which hinders their application to systems requiring light transmission, including construction, automobile windshield, and energy sectors.^[^ ^83^ ^]^ A typical example is solar cell panels which require exceptional optical transparency to maximize the absorption of sunlight and efficient conversion to electrical energy.^[^ ^120^ ^]^ In cold regions, the accumulation of ice on the surface of solar cell panels can diminish their power generation efficiency, necessitating the integration of light‐induced de‐icing capabilities. To design a coating for such applications while exhibiting multi‐functions as mentioned above, it is essential to strike a balance between the material choice, structural, optical, and thermal properties to meet the specific requirements of efficient de‐icing application.

To date, it is extremely challenging for current photothermal de‐icing material designs to meet all these requirements. Most existing solutions address only one or two of these aspects and remain at the laboratory scale. Advancing these technologies to a higher technology readiness level will require multidisciplinary collaboration among engineers and scientists. Developing the next generation of photothermal coatings that combine durability, scalability, and efficiency is critical for realizing their full industrial potential.

## Conflict of Interest

The authors declare no conflict of interest.

## Supporting information



Supporting Information
