# Supplementary material for: A Critical Perspective on Photothermal De‐Icing
Source: Adv Mater. 2024 Dec 23;37(7):2415237. doi: 10.1002/adma.202415237 (PMC11837899; doi:10.1002/adma.202415237)
Supplement: Supplementary file 1 — Supporting Information [file ADMA-37-2415237-s001.docx]

**A Critical Perspective on Photothermal De-icing**

*Siyan Yang, Jiazheng Liu, Muhammad Jahidul Hoque, Anxu Huang, Yiyang Chen, Wentao Yang, Jie Feng, Nenad Miljkovic**

S. Yang, J. Liu, M. Hoque, A. Haung, Y. Chen, W. Yang, J. Feng, N. Miljkovic

Department of Mechanical Science and Engineering, The Grainger College of Engineering, University of Illinois at Urbana-Champaign, Urbana, Illinois 61801, USA

E-mail: nmiljkov@illinois.edu

N. Miljkovic

Materials Research Laboratory, University of Illinois at Urbana-Champaign, Illinois 61801, USA

Department of Electrical and Computer Engineering, The Grainger College of Engineering, University of Illinois at Urbana-Champaign, Urbana, Illinois 61801, USA

Institute for Sustainability, Energy and Environment, University of Illinois at Urbana-Champaign, Urbana, Illinois 61801, USA

International Institute for Carbon Neutral Energy Research (WPI-I2CNER), Kyushu University, 744 Motooka, Nishi-ku, Fukuoka 819-0395, Japan

**Table S1** Ice adhesion on different surface designs (surface temperatures are from -5 to -50 ℃)

| **Surface design principle** | **Materials** | **Ice adhesion/kPa** | **Ref** |
| --- | --- | --- | --- |
| **Metals** | Aluminum | 108, 114, 124, 276, 289, 320, 320, 380, 390, 400, 450, 580, 650, 750, 780, 1360, 1670 | [1] |
|  | Steel | 250, 280, 280, 440, 450, 500, 500, 698, 1000, 1100, 1580, 2500, 2640, 2750 | [1a, 1c, 2] |
|  | Brass | 540 | [1c] |
| **Unmodified polymers** | Tecnoflon | 389 | [2b] |
|  | Polyethyl methacrylate (PEMA) | 510, 672 | [2b, 3] |
|  | Polyemethyl methacrylate (PMMA) | 280, 463, 737 | [2b, 3-4] |
|  | Polybutyl methacrylate (PBMA) | 384, 519 | [2b, 3] |
|  | Polycarbonate (PC) | 302, 400 | [2b, 4] |
|  | Cross-linked polydimethyl  Siloxane (PDMS) | 243, 291, 317 | [2b, 3, 5] |
|  | Fluorodecyl polyhedral oligomeric silsesquioxane (POSS) | 250 | [2b] |
|  | PVC | 248A | [4] |
|  | Nylon | 373 | [4] |
| **Superhydrophobic surface** | Hydrophobic aluminum | 28, 40, 125 | [6] |
|  | Superhydrophobic aluminum | 0.01, 0.07, 0.28, 1.89, 2, 4, 5, 32, 38, 48, 50, 60, 75, 80, 80, 109, 110, 680 | [1e, 6-7] |
|  | PDMS soft particles with OTS@SiO_2_ nanoparticles | 2, 26, 48, | [8] |
|  | PTFE embedded with silicon dioxide (SiO_2_) nanoparticles | 260, 500, 700, 800, 850, 1050, 1250, 1500, 1800, 1850 | [9] |
|  | Silica nanoparticles mixed with thermoplastic polyurethane | 45, 50, 110, 145, 170, 175, 210, 220, 225 | [10] |
|  | Silicone rubber | 32, 33, 34, 40, 42, 45, 72, 80, 85, 90, 100 | [7c] |
| **Lubricant-infused surface** | K100-F13-PPy-Al | 15.6 | [1d] |
|  | Silicon rubber | 30, 35, 40 | [11] |
|  | PTFE with perfluorinated oil | 8 | [12] |
|  | PTFE with silicone oil | 13 | [12] |
|  | PP with perfluorinated oil | 29 | [12] |
|  | PP with silicone oil | 33 | [12] |
|  | PP with rapeseed oil | 38 | [12] |
|  | PDMS with oil | 6.5 | [13] |
|  | PVC with oil | 5.2, 16 | [13] |
|  | PS with oil | 24, 27 | [13] |
|  | VF 40 with oil | 4.3, 4.9, 11 | [13] |
|  | CF with oil | 9, 50 | [13] |
|  | PDMS with silicone oil | 7, 7, 10, 14, 15, 18, 20, 22, 28, 37, 60, 95 | [14] |
| **Polymer brush/gel** | PDMS brush | 40, 55, 80, 145 | [15] |
|  | Crosslinked polyurea (including hard and soft segments) and silicone oil (uncrosslinked polymer chains) | 24, 42, 58, 63 | [16] |
|  | Polydimethylsiloxane | 26.1 | [5] |
|  | The combination of PU, polyurethaneanionomer, and isophorone diamine | 27, 39, 92, 253 | [17] |
|  | PDMS- PEG amphiphilic copoly-mers | 57, 60, 108, 115, 117 | [3] |
|  | Organogels contained AR20 and TSF 437 | 1 | [18] |
|  | The combination of vinyl-PDMS, hydride-PDMS, and trimethyl-PDMS | 15, 16, 20, 25, 30, 35, 40, 50 | [19] |
|  | PDMS-grafted polyelectrolyte hydrogel | 18, 20, 25, 100, 300 | [20] |
|  | Norbornene-based ﬂuorinated polymer | 3.4, 30, 90 | [21] |
|  | PDMS/SO10 | 9, 12, 15, 30 | [22] |
|  | PDMS/t-PDMS | 7, 8, 14, 26 | [22] |
|  | PDMS/v-PDMS | 15, 35, 37, 38 | [22] |
|  | PDMS/h-PDMS | 26, 28, 31, 46 | [22] |
| **Elastomer** | Combination of high and low- stiffness PDMS | 5, 6, 7, 15, 16, 17, 22, 23, 24, 25, 26, 27, 36, 37, 38, 43, 44, 45 | [23] |
|  | PDMS with underlying SUS micropillars | 160, 80, 180, 145 | [24] |
|  | SG 184 | 6, 9, 10, 14, 15, 16, 17, 18, 31, 35, 35, 37, 37, 41, 45, 46, 47, 55, 58, 64, 64, 67, 75, 87, 89, 145, 173, 173, 178, 264, 302 | [25] |
|  | PDMS sponge | 8, 9, 10, 12, 15, 18, 25, 32 | [26] |
|  | The mixture of PDMS with two different modules | 1, 2, 7, 9, 10 | [27] |
| **Low-interfacial-toughness surface** | Ultra-high molecular polyethylene | 140, 160, 230, 240, 280 | [28] |
|  | LIT PS | 141 | [4] |
|  | LIT PVC | 107 | [4] |
|  | LIT PDMS | 115 | [4] |
| **Suspended metallic surface** | Steel | 0.28, 3, 4, 5, 7, 8, 9, 11, 12, 13, 14, 15, 16, 18 | [1c, 29] |
|  | Aluminum | 0.08, 11, 7.5 | [1c] |
|  | Brass | 0.08 | [1c] |

**Table S2** Temperature rise of plasmonic localized heating designs

| **Materials** | **Temperature rise (°C)** | **Ambient temperature (℃)** | **Solar intensity** | **Illumination time (s)** | **Ref** |
| --- | --- | --- | --- | --- | --- |
| Ag-Cu | 25.43 | 20 | 1-sun | 800 | [30] |
| Al | 45 | 28 | 1-sun | 600 | [31] |
| Al-MN (interval 120 μm, MN refers to micro-nano structure) | 4 | 28 | 1-sun | 100 | [32] |
| Al-MN (interval 100 μm) | 30 | 30 | 1-sun | 300 | [32] |
| Al-MN (interval 80 μm) | 45 | 30 | 1-sun | 300 | [32] |
| Al-MN (interval 60 μm) | 47 | 30 | 1-sun | 300 | [32] |
| Al-MN (interval 40 μm) | 50 | 30 | 1-sun | 300 | [32] |
| Au-TiO_2_ | 45 | 30 | 1-sun | 300 | [33] |
| Au-TiO_2_ | 26 | 27 | 3-sun | 130 | [34] |
| CWO-MN | 45 | 23 | 1-sun | 800 | [35] |
| TiN | 60 | 25 | 1-sun | 300 | [36] |
| TiN-biochar | 62 | 0 | 1-sun | 300 | [37] |
| TiN-SiO_2_ | 70 | 25 | 1-sun | 300 | [38] |
| TiN-PDMS | 110 | 25 | 10-sun | 90 | [39] |
| TiN-PTFE | 35 | 25 | 1-sun | 100 | [40] |

**Table S3** Temperature rise of non-radiative relaxation designs

| **Materials** | **Temperature rise (°C)** | **Ambient temperature (℃)** | **Solar intensity** | **Illumination time (s)** | **Ref** |
| --- | --- | --- | --- | --- | --- |
| Cermet | 35 | -5 | 1-sun | 100 | [41] |
| Fe_3_O_4_ sponge | 80 | -30 | 1-sun | 600 | [42] |
| Fe_3_O_4_-PDMS (weight ratio 200) | 53 | 25 | 1-sun | 200 | [43] |
| Fe_3_O_4_-PDMS (weight ratio 20) | 54 | 25 | 1-sun | 200 | [43] |
| Fe_3_O_4_-PDMS (weight ratio 2) | 55 | 25 | 1-sun | 200 | [43] |
| Fe_3_O_4_-PDMS (weight ratio 1) | 57 | 25 | 1-sun | 200 | [43] |
| Fe_3_O_4_-PDMS (weight ratio 1/2) | 60 | 25 | 1-sun | 200 | [43] |
| Fe_2_O_3_ (fabrication under Ar pressure 10 Pa) | 52 | -50 | 1-sun | 1000 | [44] |
| Fe_2_O_3_ (fabrication under Ar pressure 20 Pa) | 55.5 | -50 | 1-sun | 1000 | [44] |
| Fe_2_O_3_ (fabrication under Ar pressure 30 Pa) | 58.9 | -50 | 1-sun | 1000 | [44] |
| CuFeMnO_4_ | 20 | 26 | 1-sun | 150 | [45] |

**Table S4** Temperature rise of molecular thermal vibration designs

| **Materials** | **Temperature rise (°C)** | **Ambient temperature (℃)** | **Solar intensity** | **Illumination time (s)** | **Ref** |
| --- | --- | --- | --- | --- | --- |
| CNT-Cu | 22 | 25 | 1-sun | 600 | [46] |
| CNT-Cu | 27 | 20 | 1-sun | 500 | [30] |
| CNT-1SiO_2_ | 60 | 20 | NIR irradiation | 100 | [47] |
| CNT-3SiO_2_ | 58 | 20 | NIR irradiation | 100 | [47] |
| CNT-9SiO_2_ | 47 | 20 | NIR irradiation | 100 | [47] |
| CNT-PDMS | 135 | 30 | 808 nm/NA | 25 | [48] |
| CNT-PDMS | 55 | 25 | 1-sun | 360 | [49] |
| CNT-PEGDA | 32 | 24 | 1-sun | NA | [50] |
| CNT-PEGDA | 37 | 24 | 1-sun | NA | [50] |
| CNT-PEGDA | 66 | 24 | 1-sun | NA | [50] |
| CNT-PDA | 60 | 25 | 1-sun | 180 | [51] |
| CNT-Xerogel | 70 | -30 | 1-sun | 100 | [52] |
| CWT | 75 | 22 | 1-sun | 100 | [53] |
| 0%GO-SiO_2_-3%TiC | 30 | 20 | 808 nm/2W | 40 | [54] |
| GO-SiO_2_-3%TiC | 38 | 20 | 808 nm/2W | 40 | [54] |
| GO-SiO_2_-9%TiC | 45 | 20 | 808 nm/2W | 40 | [54] |
| 0.05%rGO-PDMS | 40 | 25 | 1-sun | 200 | [55] |
| 0.5%rGO-PDMS | 50 | 25 | 1-sun | 200 | [55] |
| 0% GOF-PVDF | 10 | 20 | 1-sun | 150 | [56] |
| 1% GOF-PVDF | 20 | 20 | 1-sun | 150 | [56] |
| 3% GOF-PVDF | 30 | 20 | 1-sun | 150 | [56] |
| 5% GOF-PVDF | 35 | 20 | 1-sun | 150 | [56] |
| 10% GOF-PVDF | 50 | 20 | 1-sun | 50 | [56] |
| Graphene-PE | 26 | 40 | 1-sun | 180 | [57] |
| Carbon-PDMS-1 | 70 | 20 | 1-sun | 300 | [58] |
| Carbon-PDMS-2 | 60 | 20 | 1-sun | 300 | [58] |

**Table S5** Comparison of three photothermal mechanisms for efficient de-icing

| **Mechanism** | **Wavelength/nm** | **Solar absorbance** | **De-icing performance** | **Refs** |  |  |  |  |
| --- | --- | --- | --- | --- | --- | --- | --- | --- |
| **Plasmonic localized heating** | 300 - 2500 | > 90% | Cu-CNT: 196 s, -10 °C, 6 ± 0.5 μL ,1.5-sun  Cu-Ag: 185 s, -10 °C, 6 ± 0.5 μL ,1.5-sun  Cu-CNT: 358 s, -15 °C, 6 ± 0.5 μL, 2-sun  Cu-Ag: 270 s, -15 °C, 6 ± 0.5 μL, 2-sun | [30] |  |  |  |  |
|  | 400 - 800 | Mean absorption is a function of total film thickness | Polyvinylidene fluoride-coating, 2.4-sun  time N/A | [33] |  |  |  |  |
|  | visible spectral range | > 96% | Laser surface direct writing Al, FDTS, 190 s, -30 °C, 30 μL, 1-sun | [31] |  |  |  |  |
|  | 200 - 1100 | > 90% | Moth-eye-inspired texturing surfaces, 260 s, -15 °C, 10 μL, heated in a drying-oven | [32] |  |  |  |  |
|  | 300 - 700 | N/A | Au nanoparticles (20 ± 5 nm), 10 μL, 0.3-sun | [34] |  |  |  |  |
|  | 500 - 1000 | > 88% | TiN-PTFE film, 15 s, -10 °C, relative humidity 70 ± 5%, 25 × 25 mm^2^ area, 10-sun | [40] |  |  |  |  |
|  | 295 - 2500 | 90% | Low-emissivity solar-assisted superhydrophobic coating, 380 s, -15 °C, 10 mL, 1-sun |  |  |  |  |  |
|  | 300 - 2500 | ~90% | Superhydrophobic selective solar absorber, 67 s, -15 ± 1 °C, ice sheet, 1-sun | [36] |  |  |  |  |
|  | ultraviolet and near-infrared light | > 90% | Embedding cesium-doped tungsten trioxide and benzotriazole nanoparticles into a thin resin film, 260 s | [35] |  |  |  |  |
|  | 300 - 1300 | > 97% | TiN coating, 3s, -10 °C, 10 μL, 10-sun | [39] |  |  |  |  |
|  | 400-1400 | 93% | PMMA-iAg, N/A | [59] |  |  |  |  |
|  | 300 - 2500 | > 95% | Fluorinated biochar/TiN, 600 s, -20 °C, 1 mm-thick ice layer, 1-sun | [37] |  |  |  |  |
| **Non-radiative relaxation** | 250 - 1600 | > 91% | CuFeMnO_4_/PDMS, 12 min, -14.6 °C, 0.4~0.5-sun | [45] |  |  |  |  |
|  | Ultraviolet-visible region and near infrared region | > 91% | Condensate self-removing solar anti-icing /frosting surface, ice free even under an ambient environment of -50 °C, 1-sun | [44] |  |  |  |  |
|  | 500 - 1500 | 90% | Durably corrosive superhydrophobic coating with photothermal effect (DCSCPE), -30 °C, 1-sun | [43] |  |  |  |  |
|  | 400 - 800 | 95% | Photothermal trap, 50 – 300 s, 1.8-sun, -25 °C (*h* =17.3 W m^−2^ K^−1^), 40 μL | [41] |  |  |  |  |
| **Thermal vibration in molecules** | 300 - 850 | > 95% | (CNTs)-SiO_2_, 5 s, ice layer, -20 ℃, 10 mL, 10-sun | [47] |  |  |  |  |
|  | 280 - 2500 | PMHS-ink/PET>90%  PMHS-Ink/Ni-P/PET > 97%  Ni-P/PET>85% | PMHS-Ink/Ni-P/PET435 s ± 27 s; PMHS-Ink/PET 678 s ± 63 s; Ni-P/PET 692 s ± 77 s, -10 ℃, 10 μL, 1-sun | [60] |  |  |  |  |
|  | 380 - 2500 | 96.3% | Cu-OTS + CNT, 3s, ice layer, -10 ℃, 1 sun | [46] |  |  |  |  |
|  | 300 - 2500 | Cu-Ag & Cu-CNT > 90% | Cu-CNT: 196 s, -10 °C, 6 ± 0.5 μL ,1.5-sun  Cu-Ag: 185 s, -10 °C, 6 ± 0.5 μL ,1.5-sun  Cu-CNT: 358 s, -15 °C, 6 ± 0.5 μL, 2-sun  Cu-Ag: 270 s, -15 °C, 6 ± 0.5 μL, 2-sun | [30] |  |  |  |  |
|  | 250 - 2500 | Coated fabric > 90% | HCMT-PDMS coatings on glass, 371s; HCMT-PDMS coatings on fabric, 306s, -20 °C, 50 μL, 1-sun | [53] |  |  |  |  |
|  | 300 - 2500 | Around 98.4% | MN-PEGNs10 foam surface, < 20 s, 10 μL, 25-sun | [61] |  |  |  |  |
|  | UV–Visilbe-NIR spectrometer | ~94.2% | 130 s, -18 °C, 150 μL |  |  |  |  |  |
|  | 250 - 2500 | > 60% | Ice-templated polyethylene glycol diacrylate (PEGDA)/ CNTs (ITPC) coating, -30 °C, 1-sun  time was not mentioned | [50] |  |  |  |  |
|  | 295 - 2500 | ~96% | HPG-0.3c: -35 °C, melted completely after 500 s, 80 μL, 1-sun | [55] |  |  |  |  |
|  | N/A | N/A | 10 s, -20 °C, 10-sun | [48] |  |  |  |  |
|  | N/A | N/A | PMX surface: 26 min, -30 °C, 300 μL, 0.25-sun; 6 min, 1-sun; 4 min, 1.5-sun | [52] |  |  |  |  |
|  | N/A | N/A | DDA-PDA@CNTs_8_ coating surface: 423 s, 2 mm thick ice layer, 1-sun | [62] |  |  |  |  |
|  | 200 - 2000 | ~98%  (Al/CSL) | Al/ CSL: 126 s; Al/PDMS@CS: 269 s; Al/PDMS@CS/CSL: 77 s; Al/p-PDMS/PDMS@CS/CSL: 59 s, -10 °C, 1 sun | [58] |  |  |  |  |
|  | 200 - 2000 | ~98% | P@MNS surface: 240 s, a layer of ice thickness of ~ 3 mm, -15 °C, 1-sun | [49] |  |  |  |  |
|  | N/A | N/A | PCP: 15 min, ice with a thickness of ~2 mm, -30 °C, 1-sun | [51] |  |  |  |  |
|  | 300 - 800 | ~97%  (PC 10) | PC: 2 mm ice layer frozen, -20 °C, 0.5-sun and 1-sun | [63] |  |  |  |  |
|  | N/A | N/A | 250 s, ice layer 2.5 cm × 4.5 cm × 3 mm, -25.5 °C, 25-sun | [64] |  |  |  |  |

**Supplementary References**

[1] a)H. Memon, K. Mirshahidi, K. A. Zarasvand, K. Golovin, D. S. De Focatiis, K.-S. Choi, X. Hou, *J. Mater. Sci.* **2021**, 56, 17337; b)F. Guerin, C. Laforte, M.-I. Farinas, J. Perron, *Cold Reg. Sci. Technol.* **2016**, 121, 93; c)K. Alasvand Zarasvand, C. Pope, M. Mohseni, D. Orchard, C. Clark, K. Golovin, *Adv. Mater. Interfaces* **2022**, 9, 2101402; d)P. Kim, T.-S. Wong, J. Alvarenga, M. J. Kreder, W. E. Adorno-Martinez, J. Aizenberg, *ACS Nano* **2012**, 6, 6569; e)A. Davis, Y. H. Yeong, A. Steele, I. S. Bayer, E. Loth, *ACS Appl. Mater. Interfaces* **2014**, 6, 9272; f)M. Ruan, Y. Qiao, Y. Chen, F. Chen, B. Wang, L. Lu, Z. Yu, Z. Sun, C. Zhou, J. Liu, *J. Alloys Compd.* **2022**, 925, 166640; g)M. Huré, P. Olivier, J. Garcia, *Cold Reg. Sci. Technol.* **2022**, 194, 103440.

[2] a)H. Jellinek, *Journal of Colloid Science* **1959**, 14, 268; b)A. J. Meuler, J. D. Smith, K. K. Varanasi, J. M. Mabry, G. H. McKinley, R. E. Cohen, *ACS Appl. Mater. Interfaces* **2010**, 2, 3100.

[3] D. Chen, M. D. Gelenter, M. Hong, R. E. Cohen, G. H. McKinley, *ACS Appl. Mater. Interfaces* **2017**, 9, 4202.

[4] K. Golovin, A. Dhyani, M. Thouless, A. Tuteja, *Science* **2019**, 364, 371.

[5] L. Zhang, Z. Guo, J. Sarma, X. Dai, *ACS Appl. Mater. Interfaces* **2020**, 12, 20084.

[6] a)L. B. Boinovich, K. A. Emelyanenko, A. M. Emelyanenko, *J. Colloid Interface Sci.* **2022**, 606, 556; b)T. Bharathidasan, S. V. Kumar, M. Bobji, R. Chakradhar, B. J. Basu, *Appl. Surf. Sci.* **2014**, 314, 241.

[7] a)S. Farhadi, M. Farzaneh, S. A. Kulinich, *Appl. Surf. Sci.* **2011**, 257, 6264; b)S. Kulinich, S. Farhadi, K. Nose, X. Du, *Langmuir* **2011**, 27, 25; c)K. Maghsoudi, E. Vazirinasab, G. Momen, R. Jafari, *J. Mater. Process. Technol.* **2021**, 288, 116883.

[8] H. Cheng, G. Yang, D. Li, M. Li, Y. Cao, Q. Fu, Y. Sun, *Langmuir* **2021**, 37, 12016.

[9] D. Fu, H. Zheng, W. Sheng, X. Hao, X. Zhang, S. Chang, M. Song, *Appl. Therm. Eng.* **2024**, 244, 122732.

[10] Y. Wang, J. Zhang, H. Dodiuk, S. Kenig, J. A. Ratto, C. Barry, S. Turkoglu, J. Mead, *Cold Reg. Sci. Technol.* **2022**, 201, 103623.

[11] Q. Liu, Y. Yang, M. Huang, Y. Zhou, Y. Liu, X. Liang, *Appl. Surf. Sci.* **2015**, 346, 68.

[12] H. Niemelä‐Anttonen, H. Koivuluoto, M. Tuominen, H. Teisala, P. Juuti, J. Haapanen, J. Harra, C. Stenroos, J. Lahti, J. Kuusipalo, *Adv. Mater. Interfaces* **2018**, 5, 1800828.

[13] K. Golovin, A. Tuteja, *Sci. Adv.* **2017**, 3, e1701617.

[14] P. F. Ibáñez-Ibáñez, F. J. M. Ruiz-Cabello, M. A. Cabrerizo-Vílchez, M. A. Rodríguez-Valverde, *J. Colloid Interface Sci.* **2022**, 608, 792.

[15] Z. Zehui, W. Zelinlan, L. Guang, C. Dengke, Z. Kaiteng, Z. Yantong, C. Jichen, S. Shize, L. Xiaolin, C. Huawei, *Chem. Eng. J.* **2023**, 474, 145541.

[16] J. Li, W. Jiao, H. Jin, Q. Lu, H. Sun, Y. Yin, X. He, *Chem. Eng. J.* **2023**, 478, 147339.

[17] D. Renmei, C. Jing, Z. Yifan, W. Xupeng, C. Dapeng, S. Yanlin, J. Lei, W. Jianjun, *ACS Appl. Mater. Interfaces* **2014**, 6, 6998.

[18] C. Urata, G. J. Dunderdale, M. W. England, A. Hozumi, *J. Mater. Chem. A* **2015**, 3, 12626.

[19] D. L. Beemer, W. Wang, A. K. Kota, *J. Mater. Chem. A* **2016**, 4, 18253.

[20] Z. He, C. Wu, M. Hua, S. Wu, D. Wu, X. Zhu, J. Wang, X. He, *Matter* **2020**, 2, 723.

[21] C. Zhou, X. Zhao, X. Zhao, H. Li, S. Zhang, W. Feng, Y. Zhang, *ACS Appl. Mater. Interfaces* **2020**, 12, 53494.

[22] H. Qi, X. Lei, J. Gu, Y. Zhang, X. Gu, G. Zhao, J. Yu, *Prog. Org. Coat.* **2023**, 177, 107435.

[23] C. Chen, P. Fan, D. Zhu, Z. Tian, H. Zhao, L. Wang, R. Peng, M. Zhong, *ACS Appl. Mater. Interfaces* **2023**, 15, 6025.

[24] K. Golovin, S. P. Kobaku, D. H. Lee, E. T. DiLoreto, J. M. Mabry, A. Tuteja, *Sci. Adv.* **2016**, 2, e1501496.

[25] Z. He, S. Xiao, H. Gao, J. He, Z. Zhang, *Soft matter* **2017**, 13, 6562.

[26] Z. He, Y. Zhuo, J. He, Z. Zhang, *Soft Matter* **2018**, 14, 4846.

[27] P. Irajizad, A. Al-Bayati, B. Eslami, T. Shafquat, M. Nazari, P. Jafari, V. Kashyap, A. Masoudi, D. Araya, H. Ghasemi, *Mater. Horiz.* **2019**, 6, 758.

[28] Z. Azimi Dijvejin, M. C. Jain, R. Kozak, M. H. Zarifi, K. Golovin, *Nat. Commun.* **2022**, 13, 5119.

[29] K. Alasvand Zarasvand, C. Pope, S. Nazari, D. Orchard, C. Clark, J. Brinkerhoff, K. Golovin, *Adv. Eng. Mater.* **2022**, 24, 2200573.

[30] W. Sun, Y. Wei, Y. Feng, F. Chu, *Energy* **2024**, 286, 129656.

[31] N. Li, Y. Zhang, H. Zhi, J. Tang, Y. Shao, L. Yang, T. Sun, H. Liu, G. Xue, *Chem. Eng. J.* **2022**, 429, 132183.

[32] W. Zhao, L. Xiao, X. He, Z. Cui, J. Fang, C. Zhang, X. Li, G. Li, L. Zhong, Y. Zhang, *Opt. Laser Technol.* **2021**, 141, 107115.

[33] E. Mitridis, T. M. Schutzius, A. Sicher, C. U. Hail, H. Eghlidi, D. Poulikakos, *ACS Nano* **2018**, 12, 7009.

[34] F. Tao, J. Zheng, L. Wang, Y. Yuan, F. Wan, W. Xu, Z. Huang, S. Wang, Y. Huang, *J. Alloys Compd.* **2021**, 866, 158827.

[35] W. Li, C. Lin, W. Ma, Y. Li, F. Chu, B. Huang, S. Yao, *Cell Rep. Phys. Sci.* **2021**, 2.

[36] W. Ma, Y. Li, C. Y. Chao, C. Y. Tso, B. Huang, W. Li, S. Yao, *Cell Rep. Phys. Sci.* **2021**, 2.

[37] B. Wang, P. Yu, Q. Yang, Z. Jing, W. Wang, P. Li, X. Tong, F. Lin, D. Wang, G. E. Lio, *Mater. Today Phys.* **2022**, 24, 100683.

[38] Y. Li, W. Ma, Y. S. Kwon, W. Li, S. Yao, B. Huang, *Adv. Funct. Mater.* **2022**, 32, 2113297.

[39] B. Wang, Z. Jing, M. Zhao, P. Yu, E. Ashalley, P. Li, C. Ma, X. Tong, R. Caputo, A. O. Govorov, *Adv. Opt. Mater.* **2022**, 10, 2200168.

[40] L. Ma, J. Wang, F. Zhao, D. Wu, Y. Huang, D. Zhang, Z. Zhang, W. Fu, X. Li, Y. Fan, *Compos. Sci. Technol.* **2019**, 181, 107696.

[41] S. Dash, J. de Ruiter, K. K. Varanasi, *Sci. Adv.* **2018**, 4, eaat0127.

[42] B. Yu, Z. Sun, Y. Liu, Y. Wu, F. Zhou, *Langmuir* **2023**, 39, 1686.

[43] L. Zhang, C. Gao, L. Zhong, L. Zhu, H. Chen, Y. Hou, Y. Zheng, *Chem. Eng. J.* **2022**, 446, 137461.

[44] H. Zhang, G. Zhao, S. Wu, Y. Alsaid, W. Zhao, X. Yan, L. Liu, G. Zou, J. Lv, X. He, *Proc. Nat. Acad. Sci. U.S.A.* **2021**, 118, e2100978118.

[45] M. Wang, T. Yang, G. Cao, X. Wang, Z. Jiang, C. Wang, Y. Li, *Chem. Eng. J.* **2021**, 408, 127316.

[46] Y. Wei, S. Gao, W. Sun, X. Wu, Y. Feng, F. Chu, *Appl. Therm. Eng.* **2024**, 236, 121876.

[47] F. Zhang, D. Xu, D. Zhang, L. Ma, J. Wang, Y. Huang, M. Chen, H. Qian, X. Li, *Chem. Eng. J.* **2021**, 423, 130238.

[48] Q. Wang, A. Chen, H. Gu, G. Qin, J. Zhang, J. Xu, G. Jiang, W. Liu, Z. Zhang, H. Huang, *J. Mater. Sci.* **2021**, 56, 11723.

[49] Z. Xie, H. Wang, M. Li, Y. Tian, Q. Deng, R. Chen, X. Zhu, Q. Liao, *Chem. Eng. J.* **2022**, 435, 135025.

[50] S. Miao, X. Liu, Y. Chen, *Adv. Funct. Mater.* **2023**, 33, 2212245.

[51] Y. Liu, Y. Wu, Y. Liu, R. Xu, S. Liu, F. Zhou, *ACS Appl. Mater. Interfaces* **2020**, 12, 46981.

[52] B. Yu, Z. Sun, Y. Liu, Z. Zhang, Y. Wu, F. Zhou, *ACS Appl. Mater. Interfaces* **2021**, 13, 37609.

[53] H. Li, Y. Li, J. Wu, X. Jia, J. Yang, D. Shao, L. Feng, S. Wang, H. Song, *ACS Appl. Mater. Interfaces* **2022**, 14, 29302.

[54] D. Li, L. Ma, B. Zhang, S. Chen, *Chem. Eng. J.* **2022**, 450, 138429.

[55] C. Wu, H. Geng, S. Tan, J. Lv, H. Wang, Z. He, J. Wang, *Mater. Horiz.* **2020**, 7, 2097.

[56] Y. Tian, Y. Xu, Z. Zhu, Y. Liu, J. Xie, B. Zhang, H. Zhang, Q. Zhang, *Colloids Surf. A: Physicochem. Eng. Asp.* **2022**, 651, 129586.

[57] H. Xie, W. H. Xu, Y. Du, J. Gong, R. Niu, T. Wu, J. P. Qu, *Small* **2022**, 18, 2200175.

[58] X. Wei, J. Wei, Y. Feng, J. Wang, *Prog. Org. Coat.* **2023**, 179, 107550.

[59] P. Yao, R. Yang, Q. Sun, G. Tang, X. Liu, J. H. Pu, M. Du, *Appl. Therm. Eng.* **2024**, 242, 122490.

[60] S. Zhang, F. Zhang, Z. Zhang, G. Li, H. Fu, J. Huang, Y. Wang, Z. Lei, X. Qian, Y. Lai, *Chem. Eng. J.* **2022**, 450, 138328.

[61] H. Xie, W. H. Xu, Y. Du, J. Gong, R. Niu, T. Wu, J. P. Qu, *Small* **2022**, 18, e2200175.

[62] Y. Li, H. Li, J. Wu, X. Yang, X. Jia, J. Yang, D. Shao, L. Feng, S. Wang, H. Song, *Appl. Surf. Sci.* **2022**, 600, 154177.

[63] G. Jiang, Z. Liu, J. Hu, *Adv. Mater. Interfaces* **2022**, 9, 2101704.

[64] G. Jiang, L. Chen, S. Zhang, H. Huang, *ACS Appl Mater Interfaces* **2018**, 10, 36505.
